# Strong cooperative coupling of pressure-induced magnetic order and nematicity in FeSe

**DOI:** 10.1038/ncomms12728

**Published:** 2016-09-01

**Authors:** K. Kothapalli, A. E. Böhmer, W. T. Jayasekara, B. G. Ueland, P. Das, A. Sapkota, V. Taufour, Y. Xiao, E. Alp, S. L. Bud'ko, P. C. Canfield, A. Kreyssig, A. I. Goldman

**Affiliations:** 1 Division of Materials Sciences and Engineering, Ames Laboratory, U.S. DOE, Iowa State University, Ames, Iowa 50011, USA; 2 Department of Physics and Astronomy, Iowa State University, Ames, Iowa 50011, USA; 3 HPCAT, Carnegie Institute of Washington, Argonne, Illinois 60439, USA; 4 Advanced Photon Source, Argonne National Laboratory, Argonne, Illinois 60439, USA

## Abstract

A hallmark of the iron-based superconductors is the strong coupling between magnetic, structural and electronic degrees of freedom. However, a universal picture of the normal state properties of these compounds has been confounded by recent investigations of FeSe where the nematic (structural) and magnetic transitions appear to be decoupled. Here, using synchrotron-based high-energy x-ray diffraction and time-domain Mössbauer spectroscopy, we show that nematicity and magnetism in FeSe under applied pressure are indeed strongly coupled. Distinct structural and magnetic transitions are observed for pressures between 1.0 and 1.7 GPa and merge into a single first-order transition for pressures ≳1.7 GPa, reminiscent of what has been found for the evolution of these transitions in the prototypical system Ba(Fe_1−*x*_Co_*x*_)_2_As_2_. Our results are consistent with a spin-driven mechanism for nematic order in FeSe and provide an important step towards a universal description of the normal state properties of the iron-based superconductors.

## Abstract (web summary)

Whilst strong coupling between structure and magnetism is a signature of many of the Fe-based superconductors, no evidence for this has been reported in the normal state of FeSe. Here, the authors demonstrate strong coupling between nematicity and magnetism in FeSe under applied pressure.

## Graphical abstract

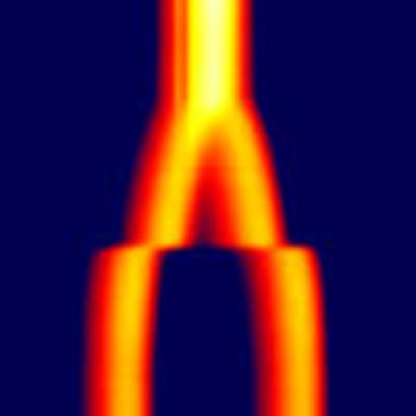

Unconventional superconductivity is generally believed to be mediated by fluctuations associated with ordering (magnetic, orbital or charge) found in the normal state of the material^1^,^2^,^3^. For the high-temperature iron-based superconductors, a leading candidate is the ubiquitous stripe-like antiferromagnetic (AFM) fluctuations associated with the magnetic ordering in the normal state. In some scenarios, these same spin fluctuations also lead to nematic electronic order, identified by a tetragonal-to-orthorhombic (T–OR) structural transition^4^,^5^. In this context, nematicity corresponds to orientational order between two AFM sublattices that are only weakly coupled because of frustration that arises from large next-nearest-neighbour magnetic interactions^6^,^7^,^8^. However, the magnetic origin of nematicity has been recently challenged by the unusual properties of FeSe^9^,^10^,^11^,^12^,^13^,^14^,^15^.

For most iron-based superconductors, including *A*Fe_2_As_2_ (*A*=Ca, Sr, Ba), *R*FeAsO (*R*=rare earth) and NaFeAs, stripe-like AFM order and nematic order, in the form of a T–OR transition, occur either simultaneously or in relatively close proximity in temperature, and both can be tuned by doping or applied pressure. At ambient pressure, FeSe undergoes a T–OR transition at a temperature of *T*_s_=90 K, but no ordered AFM state has been observed^16^. However, muon spin-resonance (*μ*SR) measurements on polycrystalline samples have demonstrated the emergence of magnetic order in FeSe under applied pressures greater than ∼0.8 GPa (refs 17, 18), and the magnetic transition temperature, *T*_m_, increases with pressure whereas the structural transition is suppressed^19^,^20^,^21^,^22^. Furthermore, superconductivity in FeSe shows a four-fold increase to a transition temperature of *T*_c_=37 K under pressure^23^, accompanying the increase of *T*_m_^19^,^20^,^22^. Taken together, the absence of magnetic order in the presence of nematic order at ambient pressure and the opposing evolutions of magnetic and nematic order with pressure, call into question the notion of a cooperative relation between magnetism and structure as the origin of the nematic order in FeSe^9^,^10^,^11^,^12^,^13^,^14^,^15^.

Electrical resistivity measurements under applied pressure provide valuable information regarding the existence of phase transitions in the pressure–temperature phase space^20^,^21^,^22^, but microscopic measurements under pressure are required to fully elucidate the underlying nature of the transitions, the associated order parameters and the relationship between the magnetism and structure of FeSe. Although direct microscopic information concerning the magnetic structure is best obtained using neutron diffraction, several previous measurements have been unsuccessful in detecting the expected small ordered magnetic moment^18^,^22^. Therefore, we have used both high-energy x-ray diffraction (HE-XRD) and time-domain Mössbauer spectroscopy through nuclear forward scattering (NFS) to study the structural and magnetic transitions in single-crystalline FeSe under pressure. Distinct structural and magnetic transitions, and a strong cooperative coupling of the respective order parameters are observed for pressures between 1.0 and 1.7 GPa. The transitions merge into a single first-order phase line for pressures >1.7 GPa. The evolution of the phase lines and the order parameters is reminiscent of the prototypical doped system Ba(Fe_1−*x*_Co_*x*_)_2_As_2_ and is consistent with a spin-driven mechanism for nematic order also in FeSe. Our results broaden the base for a universal description of the magnetic, structural and electronic properties of the iron-based superconductors.

## Results

### HE-XRD and NFS measurements

**Figure 1:**
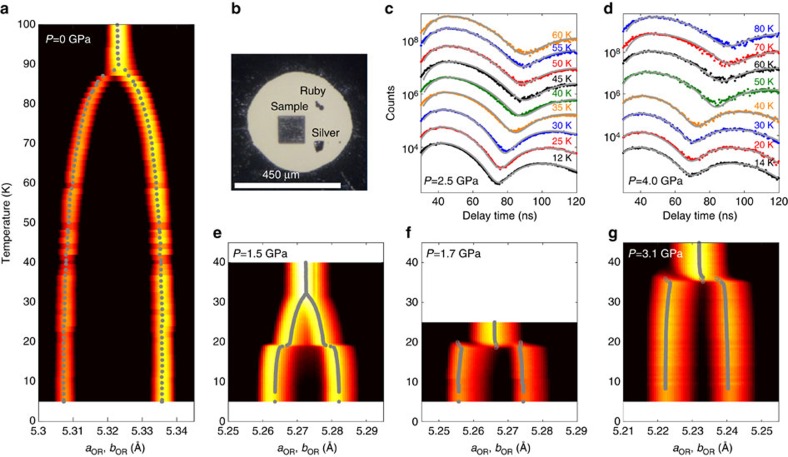
Synchrotron high-energy x-ray diffraction and time-domain ^57^Fe Mössbauer spectroscopy of FeSe under pressure. (**a**,**e**–**g**) Show the evolution of the in-plane lattice parameters at various pressures determined from the splitting of the tetragonal (*HH*0) Bragg peaks. The colour corresponds to detector intensities integrated over the transverse scattering directions. (**b**) Shows a photograph of the content of the diamond anvil pressure cell used for these measurements including the FeSe single crystal, and ruby and silver pressure indicators. (**c**,**d**) Display the time-domain ^57^Fe Mössbauer spectra at pressures *P*=2.5 and 4 GPa, respectively, with these data sets vertically offset for clarity. Grey lines are fits to the data.

The essential results of our study are summarized in Fig. 1. Figure 1a shows that FeSe undergoes a continuous T–OR transition at ambient pressure at *T*_s_=90 K. This is observed as a splitting of the (*HH*0) Bragg peaks related to the in-plane orthorhombic lattice parameters *a*_OR_ and *b*_OR_. At an applied pressure of 1.5 GPa (Fig. 1e), this structural transition is suppressed to *T*_s_=32 K. However, as the temperature is further lowered there is a subsequent abrupt increase of the splitting at 19 K. When the pressure is increased to 1.7 GPa only the discontinuous T–OR transition remains (Fig. 1f), and *T*_s_ increases to 35 K at 3.1 GPa (Fig. 1g). To correlate the features observed in the HE-XRD measurements with the evolution of the magnetism in FeSe, Fig. 1c,d show the NFS (time-domain Mössbauer spectra) at two different pressures, *P*=2.5 and 4.0 GPa. The small hyperfine field associated with magnetic ordering in FeSe is quite challenging for conventional Mössbauer spectroscopy^23^, but is readily observed in the data from our NSF measurements. The distinctive feature in these spectra is the minimum at intermediate delay times, which is determined by a convolution of the magnetic hyperfine field, quadrupolar splitting and effective sample thickness (see the ‘Methods' section). Our fits to these spectra find that the shift in the minima at ∼30 K (*P*=2.5 GPa) and 40 K (*P*=4 GPa) is attributable to the presence of a hyperfine field at these temperatures and pressures, which confirms the onset of magnetic order in FeSe under pressure.

### Coupled first-order structural and magnetic transitions

**Figure 2:**
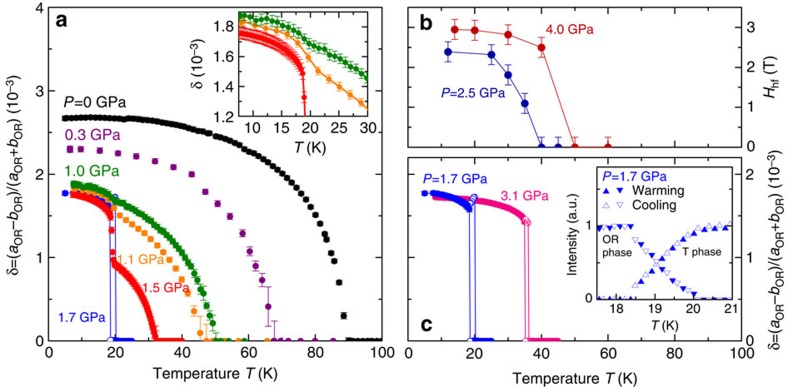
Structural and magnetic order parameters of FeSe under pressure. (**a**,**c**) Plot the orthorhombicity, 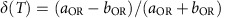, as a function of temperature at various pressures. Error bars indicate 2*σ*, the fitting error. The inset in **a**, shows a magnified view of the data close to the discontinuous change in δ near 19 K. A coexistence region for the orthorhombic and tetragonal phases is observed at pressures *P*=1.7 and 3.1 GPa as indicated by open symbols and vertical lines. The inset of **c**, shows the relative integrated intensities of the tetragonal (T) and orthorhombic (OR) phases on warming and cooling respectively, at *P*=1.7 GPa. Any thermal hysteresis is smaller than the point spacing (0.2 K), whereas the coexistence temperature region spans 1.5 K. (**b**) Displays the magnetic hyperfine field *H*_hf_ derived from CONUSS fits^37^ of the NFS data in Figs 1c,d. The error bars were estimated from fits to the data in the paramagnetic phase and include a 2% fitting error.

**Figure 3:**
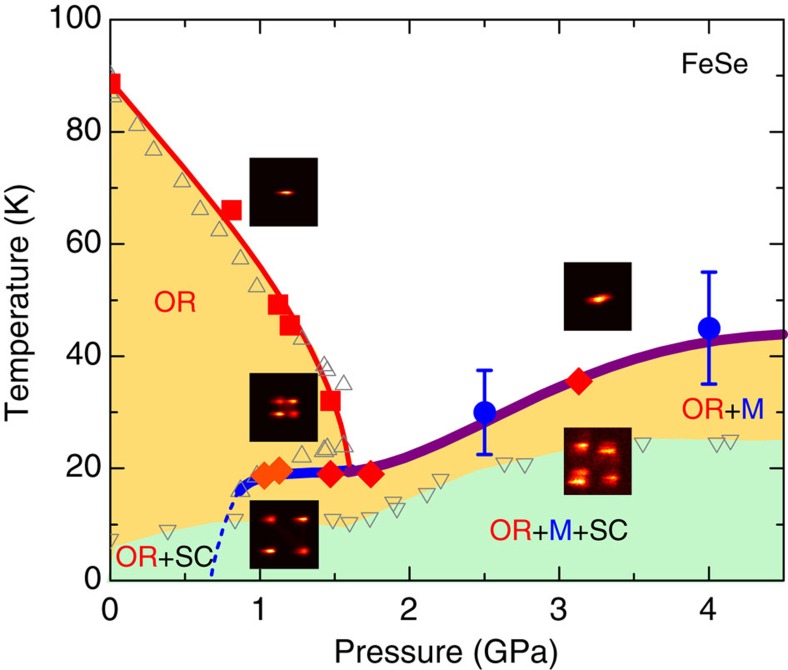
The Pressure–Temperature phase diagram of FeSe. Phase regions are labelled with the orthorhombic (OR), magnetic (M) and superconducting (SC) ordered states. The transition temperatures obtained from the present single-crystal HE-XRD measurements (red symbols) and fits of the NFS data (blue symbols, with error bars estimated from the width of the temperature steps in the measurement). The grey erect triangles denote transition temperatures inferred from the resistivity measurements^21^ on samples from the same batch used for the diffraction measurements and grey inverted triangles denote the measured superconducting transition temperature from previous work^19^. Thick lines in the figure represent first-order phase transitions and thin lines correspond to second-order phase transitions. The dashed line shows a tentative extrapolation. The insets to the figure show representative two-dimensional diffraction data in the respective pressure–temperature region demonstrating the splitting of the tetragonal (660) Bragg peak in the orthorhombic phase.

The evolution of the structural order parameter, determined from fits to the HE-XRD data, and the evolution of the magnetic hyperfine field are shown in Fig. 2 and summarized in the pressure–temperature phase diagram of Fig. 3. These data provide insight into the relationship between structure and magnetism in FeSe under pressure. At ambient pressure, the orthorhombic distortion 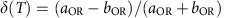 displays a smooth temperature dependence characteristic of a second-order phase transition (Fig. 2a). Starting from ∼1.0 GPa, however, we observe a small discontinuous increase in *δ* at ∼19 K (see the inset of Fig. 2a), which is very distinct at *P*=1.5 GPa and higher applied pressures, as shown in Fig. 2c. Similar measurements of the canonical BaFe_2_As_2_-type iron-based superconductors clearly established that this discontinuous increase in the orthorhombicity was associated with strong magnetoelastic coupling and a transition to stripe-like magnetic order^24^,^25^. For FeSe, the magnetic ordering temperature obtained in the *μ*SR measurements^17^ on polycrystalline samples is also consistent with the first-order structural transition measured by HE-XRD on our single-crystal sample. In Fig. 2b we show the evolution of the hyperfine field, *H*_hf_, extracted from fits to our single-crystal NFS data measured at applied pressures of *P*=2.5 and 4.0 GPa (Fig. 1c,d). The relatively small saturated values for *H*_hf_ of ∼2.5 and 3.0 T, respectively, correspond to a small ordered moment on the order of 0.2 *μ*_B_/Fe, consistent with the estimates from the previous *μ*SR experiments^18^ and explain the difficulty in detecting a magnetic signal in earlier conventional Mössbauer measurements^23^. We also find that *T*_m_ and the saturation value of *H*_hf_ increase with increasing pressure, consistent with the previous *μ*SR studies^17^,^18^. Although the density of data points is rather low, Fig. 2b suggests that the NFS spectra at *P*=4.0 GPa is consistent with a strong discontinuous transition, whereas the magnetic transition at 2.5 GPa could be described as weakly first-order.

## Discussion

Most importantly, the HE-XRD data show that the T–OR transition does not vanish above 1.7 GPa where *T*_s_, as inferred from resistivity measurements, would extrapolate to zero^13^,^20^. Rather, the abrupt onset of the orthorhombicity and the significant coexistence range of the tetragonal and orthorhombic phases (inset of Fig. 2c) demonstrate that the structural and magnetic transitions merge to a simultaneous first-order magneto-structural transition as shown in Fig. 3 and also found, for example, in the CaFe_2_As_2_ parent compound^26^. Further, our HE-XRD measurements show that the magnetic order in FeSe breaks the tetragonal symmetry of the lattice in the same manner as the ubiquitous stripe-type magnetic order in the other iron-based materials. To our knowledge, no other AFM order observed in iron-based superconductors or magnetic structures proposed for FeSe^27^ break the tetragonal symmetry in this specific way.

**Figure 4:**
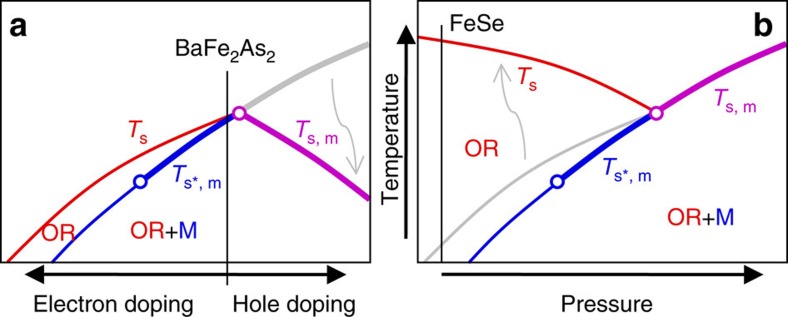
Evolution of the character of the magnetic and nematic transitions in the spin-driven nematic theory. For both panels the blue lines represent magnetic transitions, the red lines denote structural transitions and the violet lines represents the joint magneto-structural transition. The grey lines show the trends noted in the spin-driven nematic theory^5^ which differ from those found in experiments. Thick lines denote first-order transitions, whereas the thinner lines denote second-order transitions. For **a**, the control parameter is partial element substitution (doping) for the case of BaFe_2_As_2_. For **b**, the control parameter is pressure for the case of FeSe. *T*_s_, *T*_s*,m_ and *T*_s,m_ denote the structural transition temperature, the magnetic ordering temperature associated with the discontinuous change in the orthorhombicity, and the joint structural and magnetic transition temperature, respectively.

The HE-XRD and NFS results for FeSe demonstrate that all features concerning the normal state structure and magnetism, and their coupling, are very similar to other iron-based superconductors, but differ in the details of the temperature and pressure dependencies of the transitions. These results are consistent with a spin-driven mechanism for nematic ordering in FeSe. In Fig. 4, we compare the schematic phase diagrams of BaFe_2_As_2_ and FeSe with trends derived from recent theoretical studies of spin-driven nematicity^5^. Figure 4a sketches the sequence of structural and magnetic phase transitions for BaFe_2_As_2_ as a function of doping. For Co substitution for Fe in BaFe_2_As_2_ (electron doping), the magnetic and structural transitions separate in temperature and both *T*_s_ and *T*_m_ decrease with increased substitution^24^. On the other hand, for hole doping, the magnetic and structural transitions are concomitant and decrease in temperature with increased doping. The pressure–temperature diagram for FeSe in Fig. 4b also mirrors the compositional phase diagram described by Fernandes *et al*.^5^. The character of the phase transitions and their evolution with applied pressure, determined from our measurements, are consistent with this diagram, although *T*_s_ increases as pressure is lowered, in contrast to the predicted decrease. One must, however, consider the relative strengths of the interactions in such models^5^ and how these interactions evolve with pressure. In particular, a stiffening of the elastic constants with increasing pressure can result in a decrease in *T*_s_ relative to its value at ambient pressure. Similarly, an increase in the magnetic interaction, indicated by the increase in the magnetic ordering temperature and ordered magnetic moment with increasing pressure, can push *T*_s,m_ for the coupled magneto-structural transition to a higher temperature.

The origin of the large difference between *T*_s_ and *T*_m_ at low pressures remains an outstanding question, as well as the presence of nematic order in the absence of magnetic order at ambient pressure. Nevertheless, we note that this is not qualitatively different from the Ba(Fe_1−*x*_Co_*x*_)_2_As_2_ system in which there is a compositional range, close to *x*=0.06, where the nematic order is established at ∼30 K in the absence of any magnetic order^4^. Several possible explanations within a spin-driven scenario have been discussed. One possibility is, for example, that the magnetic transition is suppressed relative to the structural one via frustration^27^,^28^. However, other scenarios regarding the underlying mechanism for nematicity in FeSe have been put forward^15^,^29^,^30^,^31^ and the HE-XRD and NFS results presented here provide an important benchmark for further theoretical work.

## Methods

### Sample preparation

The FeSe single crystals were prepared using KCl/AlCl_3_ chemical-vapour transport^32^ and were thoroughly characterized in previous studies^21^,^33^. Both transition temperatures, *T*_s_ and *T*_m_, were clearly observed by resistivity measurements under pressure^21^, which facilitates a mapping of the phase diagram^20^,^21^,^22^ and supports the high-quality of our single crystals. The batch used for the HE-XRD experiment was characterized with DC magnetization and electrical resistivity measurements, and yielded sharp superconducting transitions at *T*_c_=8.7–8.8 K at ambient pressure. Samples for the NFS experiment were prepared similarly^34^ using 94% isotopically pure ^57^Fe, and had *T*_c_ values between 8.5–8.8 K.

### High-Energy X-ray Diffraction

HE-XRD measurements were performed on the six-circle diffractometer at station 6-ID-D at the Advanced Photon Source, using 100.3 keV x-rays and a beam size of 100 × 100 μm^2^. A sample with dimensions 120 × 120 × 20 μm^3^ was loaded into a membrane-driven copper–beryllium diamond anvil cell (DAC). A tungsten gasket with an initial thickness of 120 μm was pre-indented to a thickness of 70 μm, and a 660 μm hole was laser-drilled to accommodate the sample and pressure calibrants (ruby spheres and silver foil), as shown in Fig. 1b. Helium gas was used as the pressure-transmitting medium and loaded at *P*=0.5 GPa. The pressure was initially determined by the fluorescence lines from ruby spheres at ambient temperature, and, during the diffraction measurements, was determined *in situ* by analyzing selected Bragg peaks from the silver foil. The DAC was mounted on the cold finger of a He closed-cycle refrigerator and temperature-dependent measurements were performed between *T*=5 and 300 K for various pressures. The pressure was always changed at temperatures well above 120 K. Extended regions of selected reciprocal lattice planes and the powder diffraction pattern of silver were recorded by a MAR345 image plate system positioned 1.474 m behind the DAC, as the DAC was rocked by up to ±3.2° about two independent axes perpendicular to the incident x-ray beam. High-resolution diffraction patterns of selected Bragg reflections were also recorded by using a Pixirad-1 detector positioned 1.397 m behind the DAC while rocking around one of the two axes perpendicular to the x-ray beam. The images in Fig. 3 show typical examples of diffraction patterns measured using the Pixirad-1 detector, and demonstrate the excellent mosaic of the single crystal under applied pressure, as evidenced by the well-split pattern of the (*HH*0) Bragg peaks (in tetragonal notation) due to the orthorhombic distortion^35^. The orthorhombic lattice parameters were determined by fitting the Bragg peak positions after integrating the data over the transverse scattering directions. This procedure was used for both the data recorded by the Pixirad-1 detector and the data recorded by the MAR345 image plate system.

### Nuclear forward scattering

In NFS, highly monochromatic synchrotron radiation from an electron bunch excites the ^57^Fe nuclei in the sample and the decay curve is measured as a function of time. In the presence of a hyperfine interaction that splits the ^57^Fe nuclear energy levels, oscillations in the scattered intensity with time (quantum beats) are observed and can be directly compared with conventional Mössbauer spectroscopy^36^. NFS spectra were collected at beamline 16-ID-D at the Advanced Photon Source with an incident energy monochromated to the ^57^Fe nuclear resonance at 14.4125, keV, with a resolution of 2 meV, and a cross-section of 35 × 50 μm^2^ of the beam. A ^57^FeSe single crystal of dimensions 50 × 50 × 18 μm^3^ was loaded into a membrane-driven copper–beryllium DAC with 600 μm culet anvils, which allowed us to collect NFS data at *P*=2.5 and 4.0 GPa. A non-magnetic Cu–Be gasket was pre-indented to 55 μm thickness, and a hole of diameter 270 μm was laser-drilled to accommodate the sample and pressure calibrant (ruby spheres). Helium was loaded as the pressure-transmitting medium to enable hydrostatic pressure conditions. The DAC was mounted on the cold finger of a helium-flow cryostat, which achieved temperatures down to *T*=11 K. The intensity of the resonantly scattered photons in the forward direction, with 153.4 ns separation between the individual bunches, were recorded by an Avalanche Photo Diode detector. The program CONUSS^37^ was used to analyze the spectra and to determine the magnitude of the hyperfine field. The NFS spectrum at ambient temperature was well modelled without a magnetic hyperfine field, fitting only the effective sample thickness and quadrupolar splitting, which yielded a value of 0.20(5) mm s^−1^ at *P*=0.8 GPa consistent with earlier reports^23^. The low-temperature spectra were satisfactorily fit by including a hyperfine magnetic field. The two angles defining the direction of the hyperfine magnetic field with respect to the incident beam were also determined in addition to the (slightly pressure and temperature dependent) effective thickness of the sample. The values for the quadrupolar splitting were fixed to 0.15 and 0.20 mm s^−1^ at *P*=2.5 and 4.0 GPa, respectively.

### Data availability

All relevant data are available from the corresponding authors upon request.

## Additional information

**How to cite this article:** Kothapalli, K. *et al*. Strong cooperative coupling of pressure-induced magnetic order and nematicity in FeSe. *Nat. Commun.* 7:12728 doi: 10.1038/ncomms12728 (2016).
